# A Semiquantitative Framework for Gene Regulatory Networks: Increasing the Time and Quantitative Resolution of Boolean Networks

**DOI:** 10.1371/journal.pone.0130033

**Published:** 2015-06-11

**Authors:** Johan Kerkhofs, Liesbet Geris

**Affiliations:** 1 Biomechanics Research Unit, University of Liège, Liège, Belgium; 2 Biomechanics section, KU Leuven, Leuven, Belgium; 3 Prometheus, the Leuven R&D division of skeletal tissue engineering, KU Leuven, Leuven, Belgium; University of Rome Tor Vergata, ITALY

## Abstract

Boolean models have been instrumental in predicting general features of gene networks and more recently also as explorative tools in specific biological applications. In this study we introduce a basic quantitative and a limited time resolution to a discrete (Boolean) framework. Quantitative resolution is improved through the employ of normalized variables in unison with an additive approach. Increased time resolution stems from the introduction of two distinct priority classes. Through the implementation of a previously published chondrocyte network and T helper cell network, we show that this addition of quantitative and time resolution broadens the scope of biological behaviour that can be captured by the models. Specifically, the quantitative resolution readily allows models to discern qualitative differences in dosage response to growth factors. The limited time resolution, in turn, can influence the reachability of attractors, delineating the likely long term system behaviour. Importantly, the information required for implementation of these features, such as the nature of an interaction, is typically obtainable from the literature. Nonetheless, a trade-off is always present between additional computational cost of this approach and the likelihood of extending the model’s scope. Indeed, in some cases the inclusion of these features does not yield additional insight. This framework, incorporating increased and readily available time and semi-quantitative resolution, can help in substantiating the litmus test of dynamics for gene networks, firstly by excluding unlikely dynamics and secondly by refining falsifiable predictions on qualitative behaviour.

## Introduction

As molecular biology gradually shifted away from its reductionist framework towards integrative thinking and helped spawn the field of systems biology, network modelling gained more and more thrust as a pivot to formally tackle the complexity of biological systems [1]. Since the dynamical analysis of elaborate and intricate biological networks is impeded by a scarcity in kinetic information on the biochemical reactions that form them, a focus in systems biology, pioneered by the work of Kauffman [2] and Thomas [3], lies on the development of discrete and logic-based dynamical models that are better equipped to deal with the qualitative information that is typically at the modeller’s disposal. The model representations of the biochemical species and their interactions that direct biological function at the cellular scale are dubbed gene regulatory networks (GRNs), henceforth called gene networks for brevity, or protein-protein interaction (PPIs) networks. In spite of their names, both types of network often combine interactions on the gene and protein level. These qualitative models are suitable for exploratory modelling, since they do not require kinetic information or detailed mechanisms. This lack of detail also simplifies the addition of additional pathways to an existing model, potentially leading to a more global understanding of the process under investigation [4,5]. However, the relative simplicity in dynamics in the discrete models may cause them to miss behaviours that more advanced models do pick up (an example would be substrate competition), hence amounting to a clear *quid pro quo*.

Indeed, as the maxim goes in genetics, ‘no genotype is superior in all environments’ [6], neither is any model type optimal for each and every type of problem or aspect of it. The best choice of model is determined by multiple factors, including the specific research question, the type of data and available computational power. In this paper we focus on gene networks involved in cellular fate decisions, particularly in the case where no detailed quantitative information, such as time series data, is available. The model input is thus limited to topological information on the network itself and some basic information on the nature of the reactions that it represents. The type of data then lends itself to a qualitative modelling approach. The quintessential qualitative model in this context is the Boolean network model, requiring the network dynamics to be cast in ON/OFF terms. Briefly, each gene is represented by a node, that has a 0 (OFF) or 1 (ON) value for every gene. The dynamics are simulated in discrete steps using Boolean functions that describe a set of logical rules for each node [7–9]. The updating scheme determines the order in which the nodes are updated. One possible scheme is the synchronous scheme, where all nodes are updated at the same time [10].

While Boolean functions present an intuitive approach to capture interactions, they become increasingly harder to specify as the number of interaction partners of a particular gene increases, starting with the choice between AND/OR gates at 2 inputs [11]. At the same time, with the exponentially increasing number of input combinations it becomes progressively limiting to mould all output into all or nothing responses. Another issue arising in Boolean modelling is that the updating scheme often disregards qualitative information on time aspects that is intrinsic to the type of interaction between two nodes. Given that this type of information is readily available, it may prove advantageous to incorporate in the gene network model, albeit at the cost of increased complexity.

Over the years, many qualitative modelling approaches have been developed to address these two limitations [7,12,13]. Firstly, several approaches were employed to ameliorate the all or nothing response. Shumelevich et al. developed probabilistic boolean networks, which allow for multiple Boolean functions, each associated with a probability, to determine the activity of a node [14,15]. In ChemChains, continuous signals are converted to a ratio of active/inactive states in a sliding window [16]. Another avenue is the introduction of multi-value variables, allowing for multiple discrete levels rather than two in the Boolean case [17,18]. Software tools supporting multi-value variables include GINsim [5,19], Cell Net Analyzer (CNA) [20] [21], BoolNet [22] and BooleanNet [23]. An alternative methodology capable of modelling graded responses that has seen use in gene network modelling is fuzzy logic [24,25]. Fuzzy logic allows for specification of discrete categories interacting through logical rules in a continuous framework [25]. Cell Net Optimizer (CNO) is a software tool able of training, among others, constrained fuzzy logic models to data [26,27]. A variety of methods have been proposed to make the response of a Boolean network fully continuous, essentially converting Boolean functions to their continuous counterparts [28–32]. Of these, the framework of piece-wise linear differential equations has been implemented in BooleanNet [23] and Genetic Network Analyzer [33]. The MATLAB toolbox Odefy can be used to convert Boolean models to logic-based ordinary differential equations (ODEs) [34]. Likewise, SQUAD automatically derives a continuous dynamical system from a network topology and allows some parameters of the system to be tweaked [35].

Secondly, several adaptations to the standard Boolean framework augment the time resolution. A first strategy is to fine-tune the update rules. For instance, priority classes, introduced in GINsim, achieve this by enforcing that nodes in a higher priority class are updated first [36]. A similar strategy is also implemented in CNA, BoolNet and BooleanNet [20,22,23]. Another strategy is to explicitly associate a delay parameter (and possibly a noise term) to each node that specifies the timing of the node change on a (generic) time scale [37]. Alternatively, time resolution can be implicitly added by associating node updates with a certain probability, as was done in Stoll et al. [38] where a form of the Gillespie algorithm is applied to the state transition graph. As stated above, these improvements come at the cost of extra complexity, in the form of extra parameters or modelling options [7].

In this paper we introduce a discrete dynamical framework that addresses the aforementioned issues arising in the Boolean framework. The all or nothing response is mitigated by introduction of continuous normalized values in combination with an additive approach. Qualitative information on time aspects is included by dividing the model interactions into two separate speed classes. The increased model complexity permits us to capture a wider range of the system’s behaviour then would otherwise be possible in a standard Boolean model. We show that the incorporation of these features can yield new insights into the behaviour of the system.

To illustrate that the increase in the model’s complexity is justified by an increased scope of possible behaviour, we apply it to two gene networks, the first driving differentiation of cartilage cells (chondrocytes) in the growth plate (derived from [18]) and the second controlling differentiation of T helper (Th) cells [31]. Both networks are characterized by two opposing groups of transcription factors that enact distinct cell fates. For the first network, the chondrocyte differentiation drives the growth of long bones first by transformation to a proliferating phenotype followed by a further transformation causing the cell to enlarge (hypertrophy). The network examines the genetic drivers of this phenotypic switch from proliferation to hypertrophy, the former characterised by activity of Sox9, the latter by Runx2 [39]. The second network models differentiation of naïve T helper cells into two distinct lineages, Th1 and Th2, with respectively T-bet and GATA3 as ‘master’ transcription factors [40]. Of course, as the networks clearly indicate, many factors associated with these transcription factors also carry out an important role.

## Materials and Methods

**Fig 1:**
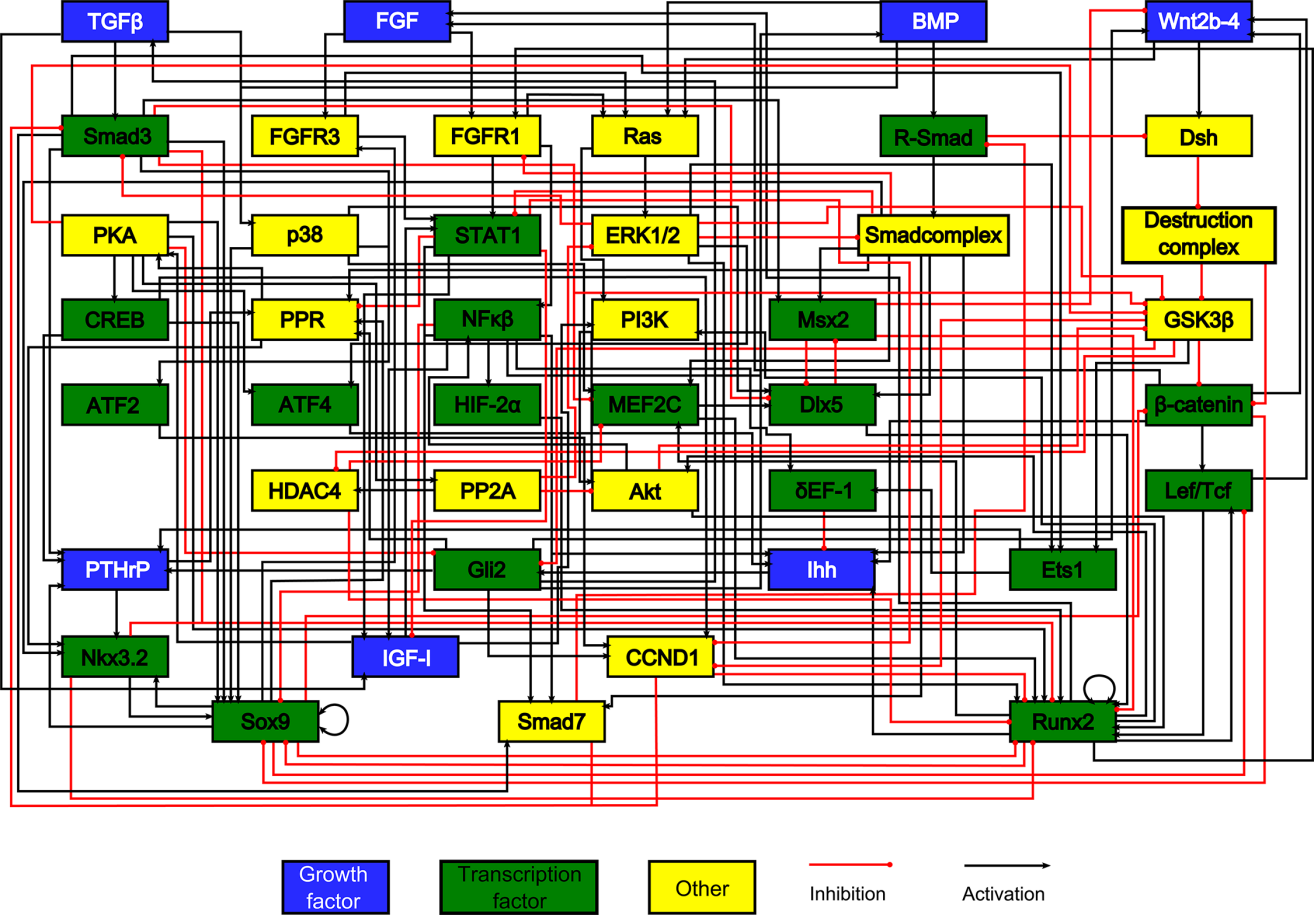
The chondrocyte gene network. Every box represents a gene, its protein or in some cases a complex of them. The interactions are represented by red and black lines if they are inhibitory and stimulatory, respectively. Blue boxes denote growth factors, green boxes are transcription factors, yellow boxes do not belong to either category. Based on [18].

**Fig 2:**
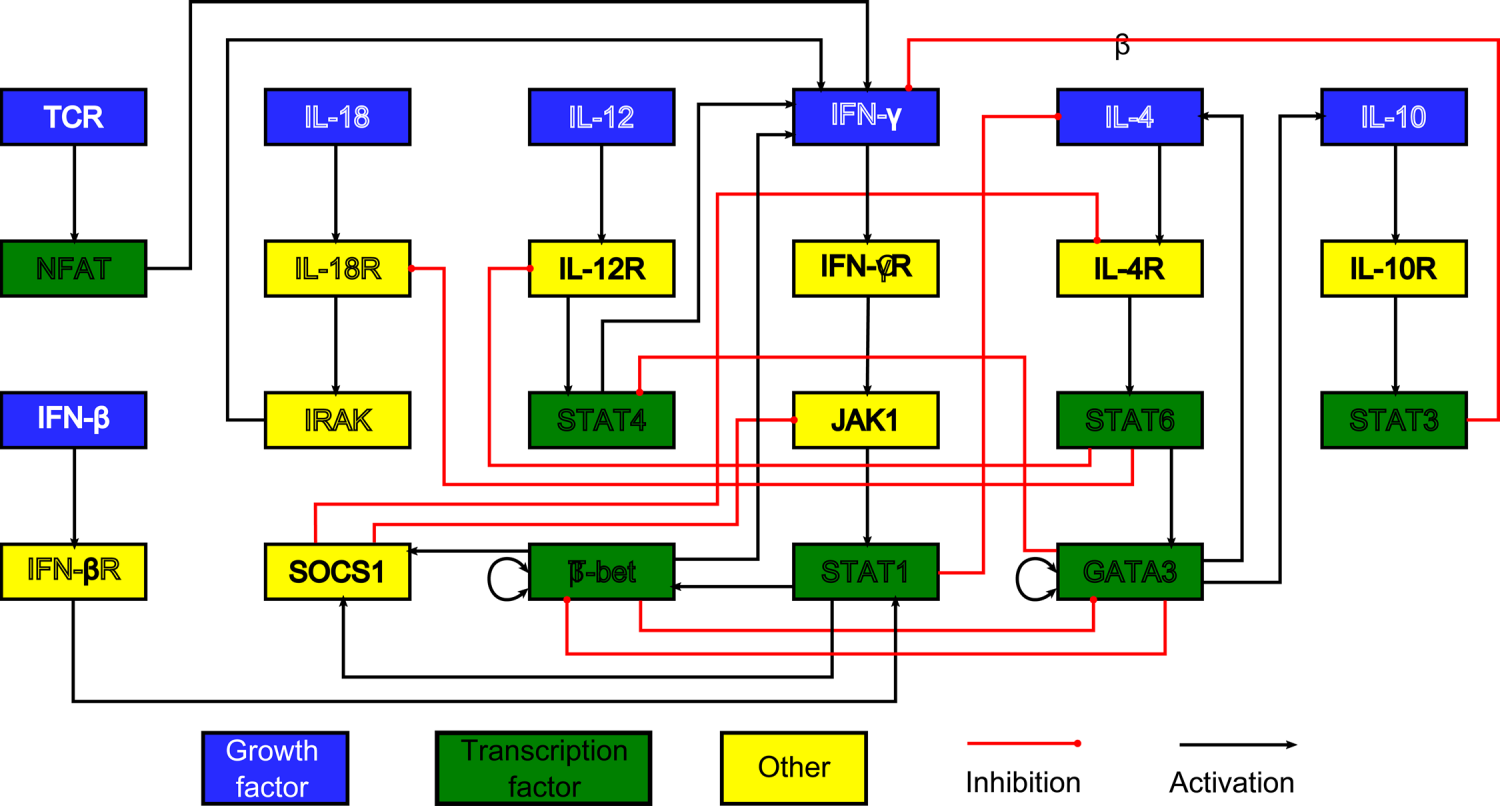
The T helper cell network. Every box represents a gene and its protein. The interactions are represented by red and black lines if they are inhibitory and stimulatory, respectively. Blue boxes denote growth factors, green boxes are transcription factors, yellow boxes do not belong to either category. Based on [31].

The chondrocyte model is based on an extensive literature study and is an expansion of a model we previously published [18]. Note that the network is an abridged version in the sense that only biological factors that receive multiple inputs are included whereas factors that simply act as a relay are omitted. For example in the linear pathway sequence Wnt to Frizzled (Fz) to Dishevelled (Dsh), Fz is not included in the model since no interactions of other pathways impede on this protein. Fig 1 is a graph depiction of the network. An overview of the equations can be found in S1 Appendix. In addition, the topology of the network is available in SBML format (S1 File). The T helper cell network was constructed from a literature study, and was investigated using discrete and continuous modelling [31,35,41]. Recently, the model has been expanded to include more lymphocytic cell types [42]. A depiction of this network is found in Fig 2. The equations describing the network can be found in S1 Appendix.

Essentially, any mathematical model of a gene network relies on two distinct parts, the representation of the topological information in the form of a directed graph, and a framework for the simulation of the dynamics of the network, for which a variety of methods is available [43–45].

Formally, a directed graph *G*(*V*, *E*) consists of a set of nodes (or vertices) V and a set of edges (or arcs) E. Each edge is an ordered pair, giving the source node and the target node [46]. In the context of a gene network, nodes represent biological factors and edges represent regulatory interactions between them. The outgoing edges indicate which factors are influenced by the node, whereas incoming edges specify upstream control, thus indicating the flow of information in the network. Growth factors, transcription factors and other biological factors are represented by blue, green and yellow boxes respectively. Graphical representations of the edges give an indication of the nature of the regulatory interaction. These interactions can be either inhibitory, in which case they are shown by a red line ending with a dot, or activatory, in which case the edge will be drawn as a black triangular line. This graph hence constitutes a topological model of the gene network, in itself devoid of dynamical information.

### Temporal resolution

As mentioned above, the presented framework differs from a classic Boolean approach in two important ways. A first difference is the employed updating mechanism. There are several ways in which the discrete updating of the nodes can be executed. One possible method is synchronous updating, where all nodes are updated simultaneously based on the value of the nodes at the previous time instance. This approach offers an important computational advantage in simulating larger networks [47]. However, the inherent assumption that all interactions have equal rate is not valid and a poor approximation in the majority of biological systems. Moreover, the introduction of an artificial external clock often gives rise to spurious dynamics [48]. For this reason we focus on asynchronous updating, where each node is updated in turn based upon either the reaction rate or other criteria in absence of this information [11].

**Fig 3:**
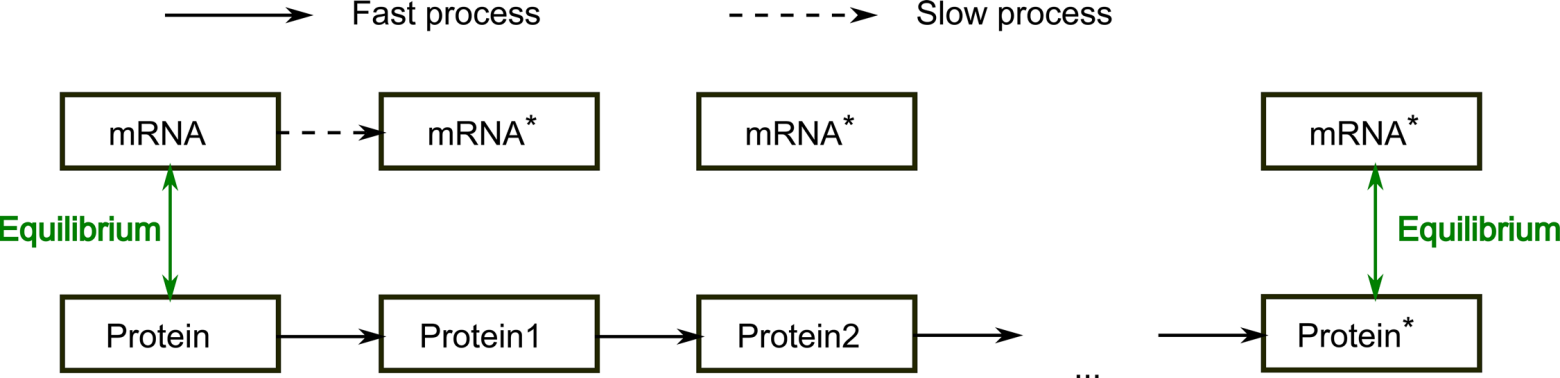
Graphical representation of the updating scheme. Every node consists of two variables, denoting the slow and fast processes, here dubbed mRNA and protein respectively. Every time the slow variables are updated, the fast variables are randomly selected to update until equilibrium is reached. Then, another slow variable is randomly updated repeating the process until overall equilibrium is attained.

While exact quantitative information is not readily available in literature, qualitative information on the system kinetics usually is, often implicit in the nature of the interaction. In the absence of accurate time information we split reactions into two priority classes, dividing the fast and slow reactions [20,36]. Priority classes were first introduced in GINsim, where each node (rather than an interaction as is the case in this study) can be categorized to a priority class [19]. The introduction of priority classes ensures that when the option is available to update both a slow and a fast reaction, the fast reaction will always be updated first. This is achieved by the allocation of a slow and fast variable to each node. These variables roughly represent transcription, translation and degradation for the slow variables, and all post-translational modifications, such as phosphorylation, as well as complex formation for the fast variables. Fig 3 is a graphic representation of the updating scheme, with one slow variable being updated randomly, followed by random updates of fast variables until the system reaches equilibrium, when no more fast variables can be updated (to within a tolerance (see below)). ext, a slow variable is randomly updated again. Overall equilibrium is reached when no variables can be updated to within a tolerance: the system has reached an attractor. The slow reactions (usually transcription) are consequently a measure for the mRNA (and protein) concentration and the fast ones determine the activity of the produced protein. Together, they determine the final activity of the node.

### Quantitative resolution

The functions that assign values to the nodes mark a second important difference between the presented framework and classic Boolean networks. In order to simulate the dynamics, a control function must be attributed to each of the nodes, assigning a value to the respective node in the next time step based on the values of all incoming interactions. In a logical framework these signals are combined through the logical operators, namely the AND, OR and NOT gates, which gives rise to some specific limitations. In the presented framework, we have opted for a more uniform additive approach with normalized and continuous values for each node which alleviates some of these limitations.

The eponymous feature of the Boolean framework is the discretization of the variables, which limits the possible outcomes of the model. Even in the absence of quantitative information, this discretization can become limiting in the resolution of the control functions, and increasingly so as the number of inputs increases. Instead of the logical discretization we have therefore adopted a semi-quantitative approach that uses normalized values to represent each node, and is able to differentiate between changes in input that would not result in a change of the discretized value. The following example will clarify this, bearing in mind that this example is valid only for the situation where no overriding biological knowledge is available, for instance establishing the dominance of the inhibition. Take a gene that has 3 activators and 1 inhibitor and is modelled as a Boolean variable determined by a threshold function (i.e. using additive logic). If we assume all interactions to be of equal strength, the situation where 3 activators are active and the situation where 2 activators and 1 inhibitor are active will not be different, while common sense dictates that, given no information on saturation or thresholds being available, the former situation will result in higher expression than the latter. The usage of normalized and continuous values circumvents this problem by adding a grey scale to the black and white Boolean (logical) approach.

We determine this continuous value for each node through the use of additive control functions, meaning that we add or subtract (for the respective cases of activating and inhibitory interactions) the values of the incoming interactions to determine the value of the node in the next time step. Since these values are normalized, the value of this sum is saturated if above one or below zero.

The value of each variable (z^s^ or z^f^ for respectively the slow and fast variables) at the next time step consequently is:

$$z_{i}^{s,f}(t+1)=\{\begin{array}{ccc} \text{1} & \text{if} & \sum_{j=1}^{N}c_{ij}z_{j}(t)\geq 1\\ \sum_{j=1}^{N}c_{ij}z_{j}(t) & \text{if} & 0<\sum_{j=1}^{N}c_{ij}z_{j}(t)<1\\ \text{0} & \text{if} & \sum_{j=1}^{N}c_{ij}z_{j}(t)\leq 0 \end{array}$$ (1)

with $z_{j}(t)=z_{j}^{s}(t)z_{j}^{f}(t)$.

Here z is a vector containing the activities for all nodes and z^s^ or z^f^ refer to the slow and fast variable respectively. N is the number of nodes in the network. *c*_*ij*_ represents the weight attributed to edges from node j to node i. As explained above, a slow and fast variable is attributed to each node. The node’s activity z is then determined by the product of the fast and the slow variable (z = z^f^ x z^s^). The value of each of these is regulated individually by an additive function. If no interactions regulate a particular variable (e.g. a node has no fast interactions), we will assume a constitutive activity, resulting in the variable’s value always being one.

**Fig 4:**
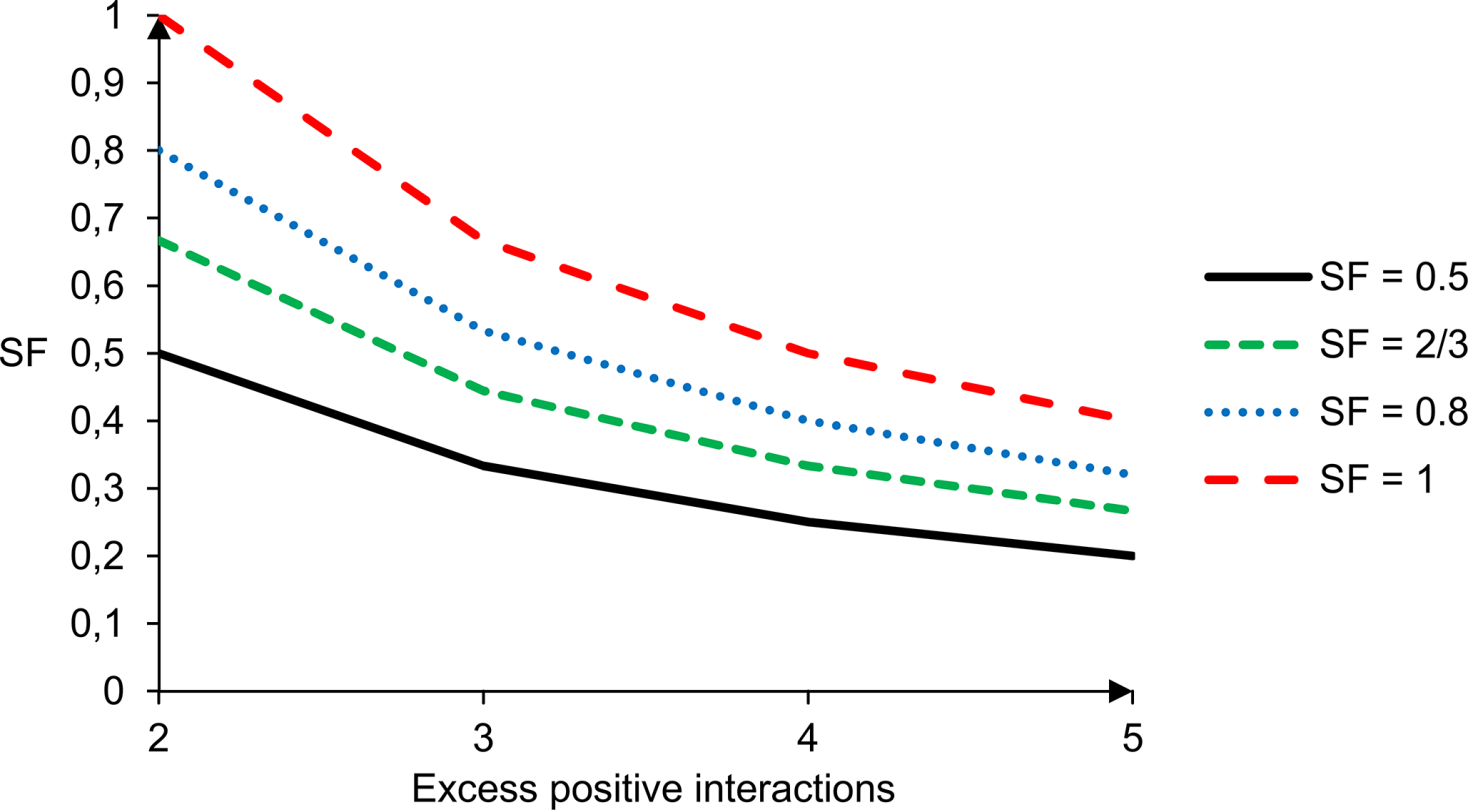
Weights for various values of the saturation factor. The value of the horizontal axis shows the number of excess positive interactions. E.g. 5 corresponds to the case where 5 positive interactions are present (or 6 positive interactions and 1 inhibition etc). When SF = 0,5 there is no saturation. For example, all 5 positive interactions must be fully active to achieve the maximal activity of 1. Alternatively, the plot can be regarded as the activity a node will take when only one upstream node, out of 2, 3, 4 and 5 nodes respectively, is active. The higher the saturation factor, the higher this value will be.

The additive sum includes a weight (c) for each term that assumes a limited amount of saturation whenever there is a majority of stimulatory interactions. In a directed graph, edges and nodes can be assigned certain labels [49]. For edges, the labels define the interaction sign *s*_*ij*_, which can be 1 (activation) or -1 (inhibition). As we assume that no additional information on interaction strengths is available, we will determine the values of *c*_*ij*_ based solely on the interaction signs:

$$c_{ij}=\{\begin{array}{ccc} s_{ij} & \text{if} & \sum_{j\in U}^{}s_{ij}\leq 1\\ 2s_{ij}\frac{SF}{\sum_{j\in U}^{}s_{ij}} & \text{if} & \sum_{j\in U}^{}s_{ij}>1 \end{array}$$

where U is the set of indexes of nodes connected to node i by an edge and SF is a saturation factor. If the positive interactions do not outnumber inhibitory interactions by more than 1, all weights are set to 1. If the number of excess positive interactions is higher than 1, we introduce a saturation factor whose value will determine how fast the node will saturate. A saturation factor SF of ½ corresponds to an absence of saturation. Higher values of SF will allow the node to saturate more rapidly, as reflected in higher weights. Fig 4 shows the weights for various values of SF and $\sum_{j\in U}^{}s_{ij}$(the number of excess positive edges). The value of SF can be seen as the activity that a node has when half of the excess positive interactions are active. For instance, in a node controlled by two positive interactions, the activity of the node will equal SF when one interaction is active (i.e. one of the upstream node’s activity is 1 and the other’s is 0).

The SF can be used to tune the network. The two networks discussed in this work have no input nodes (no node without upstream control). Since the control function for variables (see Eq 1) includes no constant term, a trivial solution (where all variables are 0) always exists in a network without input nodes. Lower values of SF will result in the dynamics of more initial conditions reaching this trivial solution. For instance, with SF = 0,5 a node with a certain amount of positive interactions would only reach its maximal value when all upstream nodes have maximal activity. Given the high degree of interconnectedness in biological networks and the numerous resulting feedback loops, this situation is increasingly unlikely as the number of upstream nodes increases and would seldom occur in highly connected nodes. As a consequence the value of these nodes would be low in most cases, increasing the likelihood of reaching the trivial solution, which is not of direct interest. A corollary would be that any non-trivial solutions would have rather low values and higher values would not be reached. This is to be avoided since the trivial solution does not necessarily have any direct biological relevance. Hence values of SF > 0,5, a limited amount of saturation, could consequently yield results closer to biological reality. On the other hand, with high values of SF, activities of nodes would saturate rather quickly. This deemphasizes the trivial solution as desired, but at the cost of the ability to discriminate when inputs are high. The choice of the SF hence allows compromising between the ability to discriminate between different situations leading to lower activities overall on the one hand, and the ability to saturate signals to higher activities, decreasing in chance to reach the trivial solution, on the other.

In the frequently arising case of 1 activator and 1 inhibitor, the value of the nodes determines potential dominance of the inhibitory interaction. Particularly, dominant inhibitory interactions have a weight of 1 and non-dominant inhibitory interactions a weight of 1/2. An example of non-dominant inhibition is found in the repression of Receptor-Smad (R-Smad) by Disheveled (Dsh) [50]. The activity of a complex is simulated by multiplying the activities of its components, as for example the combined activity of histone deacetylase 4 (HDAC4) and Smad3 [51]. In general, any dominant negative interaction should have a weight equal to that of all the activators combined. Conversely, if inhibitory interactions are more numerous, an auxiliary node can be introduced to combine their inputs into a single signal. Overall, while these rules cover a standard situation, the weights allotted to individual reactions offer a great deal of flexibility, and incorporation of relevant biological facts should always trump these *a priori* rules. As such, some adaptations to the weights in the chondrocyte network were introduced to better reflect natural expression profiles (see S1 Appendix).

### Model analyses

To find the attractors in the respective gene networks a Monte Carlo analysis, sampling and simulating random points in the state space, was performed. Since only a small percentage of the state space is sampled in this way, the possibility exists of missing an attractor in the analysis. However, we are only interested in the mainstay behaviour of the model, and as such in the attractors with large attractor basins, that dominate the system’s state space and its long term behaviour. Any attractors with very small attractor basins, that may be missed by this analysis, are unlikely to be biologically relevant.

At the same time, the Monte Carlo analysis can provide an estimate of the phenotypic robustness or canalisation of the attractor [52,53]. Waddington introduced canalisation as the ability of a cell type to stably arise from uncertain conditions [54]. In the context of discrete modelling this amounts to estimating the size of the attractor basin, as this determines the probability that any random initial state will end up in this attractor. Hence the percentage of random samples in the Monte Carlo analysis that flows into the attractor constitutes a measure of canalisation. Similarly, by setting nodes to a constant value their individual impact on the canalisation can be assessed by gauging their influence on the size of the attractor basin.

All simulations were performed in MATLAB (The Mathworks, Inc). Starting from an initial state (either in the attractor for growth factor perturbations or a random initialisation to assess the size of the attractor basin) nodes are updated according to the updating scheme shown in Fig 3. Dynamic simulations are considered to be converged when the difference between successive states is smaller than 10^−4^ for all variables. Higher tolerances do not give different results, but simulations take slightly longer to converge. Additionally, we detect spurious attractors when tolerance is set at 10^−2^. The tolerance was hence selected as a compromise between increased computational cost and avoiding spurious attractors. MATLAB files used to perform these simulations are supplied in S2 File. All simulations were done in triplicate to supply a standard deviation.

For the Monte Carlo analysis with random initialisation, assessing the size of the attractor basin, 3000 runs per simulation (9000 in total) are carried out. We selected this amount of runs to limit the standard deviation to about 1%. For the growth factor perturbations 100 runs per growth factor per simulation (300 in total) are carried out. For the Monte Carlo analysis with random initialisation only viable starting states, i.e. those states that the system can reach dynamically without intervention, are selected. This means that the activity for each node is drawn uniformly out of the interval [0,1]. The slow and fast variables are then set to reflect this activity. If the node is subject to both slow and fast control, the initial conditions of the fast and slow variable are the square root of this value. If control is uniquely fast or slow, this variable also takes on the activity value whereas the other variable is set to 1. An example of a non-viable state would be a fast node where the slow variable is not 0, which cannot occur without intervention. The Monte Carlo analysis of the attractor basins is similar to the algorithm included in the BN/PBN toolbox by Lähdesmäki and Shmulevich [14]. For the growth factor perturbations, the activity of the respective growth factor is held fixed at a perturbed value and a Monte Carlo analysis is performed with each run starting in the attractor. To illustrate the computational cost of the introduction of slow and fast variables we performed a Monte Carlo analysis of 10000 runs using the chondrocyte network, and compared it to the performance of a model without the distinction. On a 2.4 GHz processor the former took 3.7 hours, compared to the control of 1.3 hours, amounting to about a 3-fold increase in cost.

## Results

The Monte Carlo algorithm can determine the attractors for both networks. For the chondrocyte gene network, the Monte Carlo analysis indicates that three stable states exist, one of which is the trivial state (i.e. trivial solution). As expected, of the other two states one is characterized by Sox9 and the other by Runx2. The exact values of the attractors are given in S1 Table. The trivial state might be considered as out of the model’s scope and could potentially represent progenitor cells, other differentiation paths (e.g. adipocytes) or even apoptosis.

For the Th network, the attractors are given in Table 1. In this case, many attractors exist. One attractor is characterized by GATA3 expression and represents Th2 cells. The rest of the attractors have T-bet activity and consequently represent the Th1 cell type. The multitude of attractors arises because self-activation of T-bet is the only active interaction for T-bet in the attractor, meaning that any activity of the node will be stable. GATA3 also has self-activation but an additional positive feedback loop through IL-4, Il-4R and STAT6 ensures that the activity of GATA3 is 1. A similar feedback loop exists in the T-bet attractor but it is rendered inactive by the inhibition of JAK1 by SOCS1. Hence the possible attractors containing T-bet are summarised in Table 1 (by representing the value of T-bet by x). The lowest possible value, zero, corresponds to the trivial state. In practice however, the lower values of this interval are never reached. For example, no T-bet stable state is found where the activity of T-bet is below 3% (see S1 Fig). Effectively, the trivial state is a marginally stable attractor in this model, since any perturbation in T-bet will lead to a new attractor. Importantly, this ‘smearing out’ of the attractor has little effect on the canalisation of the Th1/Th2 states, and we can regard the continuum of attractors in T-bet as the Th1 cell type. A comparison with stable states detected in Mendoza and Xenarios [31] can be found in S1 Appendix.

**Table 1:** The stable states of the Th network.

**Node**	**TCR**	**NFAT**	**IFN-β**	**IFN-βR**	**IL-18**	**IL-18R**	**IRAK**	**SOCS1**
** **	**T-bet**							
**Activity**	0	0	0	0	0	0	0	2x/3
**Protein**	1	0	1	0	1	0	0	2x/3
**PTM**	0	1	0	1	0	1	1	1
** **	**GATA3**							
**Activity**	0	0	0	0	0	0	0	0
**Protein**	1	0	1	0	1	0	0	0
**PTM**	0	1	0	1	0	1	1	1
**Node**	**IL-12**	**IL-12R**	**STAT4**	**T-bet**	**IFN-γ**	**IFN-γR**	**JAK1**	**STAT1**
** **	**T-bet**							
**Activity**	0	0	0	x	4x/9	4x/9	0	0
**Protein**	1	0	0	x	1	4x/9	1	1
**PTM**	0	1	1	1	4x/9	1	0	0
** **	**GATA3**							
**Activity**	0	0	0	0	0	0	0	0
**Protein**	1	0	0	1	1	0	1	1
**PTM**	0	1	1	0	0	1	0	0
**Node**	**IL-4**	**IL-4R**	**STAT6**	**GATA3**	**IL-10**	**IL-10R**	**STAT3**	
** **	**T-bet**							
**Activity**	0	0	0	0	0	0	0	
**Protein**	1	0	0	1	1	0	0	
**PTM**	0	1	1	0	0	1	1	
** **	**GATA3**							
**Activity**	1	1	1	1	1	1	1	
**Protein**	1	1	1	1	1	1	1	
**PTM**	1	1	1	1	1	1	1	

x can in principle be any value in [0,1]. The first row gives the activity of the node. This activity is composed of the slow variable (second row), which gives the influence of the slow processes leading to protein formation, and the fast variable (third row), giving the influence of post translation modifications (PTMs).

Next, we demonstrate how the additional features in our framework, the introduction of priority classes and the additive control combined with normalised activities, broaden the scope of behaviour that the model can successfully capture/investigate. We also show that this advantage is context-specific, since for some networks the effects will not be worth the extra computational cost.

To illustrate the advantages of normalized continuous values, the first of two major differences with standard Boolean modelling, we perform a perturbation analysis on the growth factors in the network. It is a long standing tenet in developmental biology that timing and dosage are crucial considerations to determine a growth factor’s impact on differentiation, hence the need for network models to allow for examination of these effects. In terms of the gene network the first factor (timing) is equivalent to the network state at the moment the signal is received, and dosage determines how effectively the perturbed node’s outgoing edges are activated. The continuous activities of the nodes allow for a straightforward dissemination of the effect of dosage, summarised in Table 2. The chondrocyte network has 2 mutual inhibition motifs between Sox9 and Runx2 as well as between Dlx5 and Msx2. These mutual inhibitions of the network induce 2 competing attractors in the network, one with Sox9 active and another with Runx2 active [55]. Given this nature of the network, a drastic change in Sox9 or Runx2 induced by a growth factor indicates that a change to a different attractor has taken place (Runx2/Dlx5 active vs. Sox9/Msx2 active vs. neither active). By contrast, mainly through direct effects (see discussion for examples), a factor may change the activity of genes without effecting a change in attractor. In other words, the factor may change the activity of any gene (including Sox9 and Runx2) without Sox9 or Runx2 going from inactive to active or vice versa. Crucially, specifically in the case of Sox9 and Runx2, a pure Boolean network cannot pick up this relative change in activity.

**Table 2:** Perturbation analysis for growth factors in proliferating chondrocyte.

Sign:	+	+	+	+	-	-
Factor:	Wnt	Ihh	FGF	BMP	IGF	PTHrP
1%	100%	100%	100%	100%	100%	100%
	0,89	0,90	0,90	0,91	0,90	0,90
5%	100%	100%	100%	100%	100%	100%
	0,85	0,91	0,89	0,93	0,90	0,90
10%	100%	100%	100%	100%	100%	100%
	0,80	0,92	0,89	0,97	0,90	0,90
15%	100%	100%	100%	100%	100%	100%
	0,74	0,92	0,89	1,00	0,90	0,90
20%	0%	100%	100%	100%	100%	100%
	0	0,94	0,88	1	0,90	0,90
30%	0%	100%	100%	99% ± 0,6%	100%	0%
	0	0,95	0,87	1	0,16	0
40%	0%	100%	100%	45% ± 6%	100%	0%
	0	0,97	0,87	1	0,10	0
50%	0%	100%	99% ± 0,6%	1% ± 1%	100%	0%
	0	1	0,86	1	0,06	0

For the six growth factors present in the model, the effects of increasing perturbations are tabulated. The upper number in each cell indicates the percentage of runs that remains in the attractor (i.e. the fraction of perturbations that does not result in a switch to a different attractor). The lower number in each cell indicates the activity of Sox9 in the attractor. The wild type activity of Sox9 is 0,9. The simulations were done in triplicate. When relevant, the standard deviation is given.

**Fig 5:**
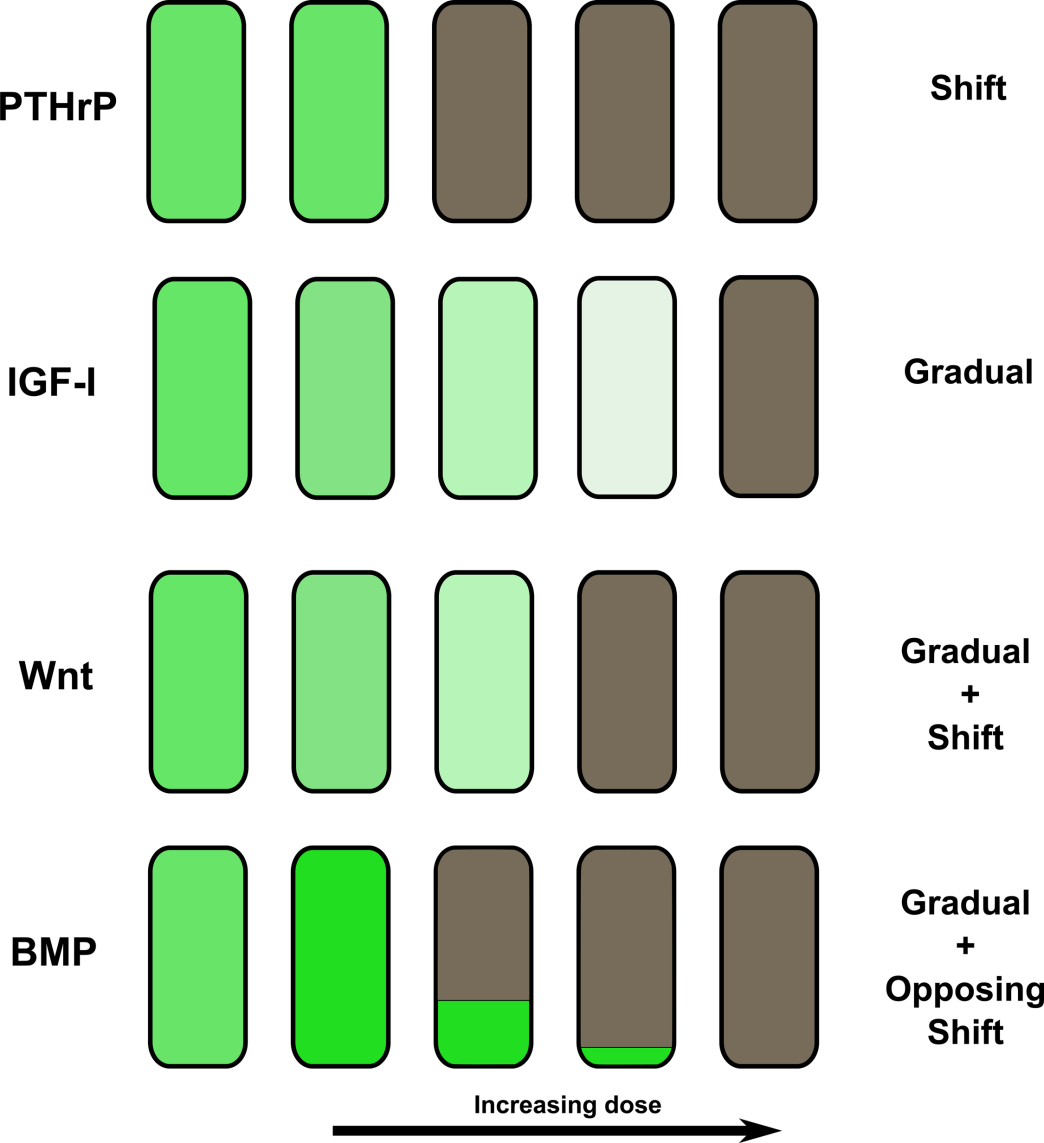
Qualitatively different outcomes of increasing activity. The dose response of the network to an increase in the activity of a growth factor can show different outcomes depending on the growth factor. Attractors are colour-coded with green standing for attractors with Sox9 activity and grey for other attractors. The tint gives an indication of Sox9 activity. Dark green indicates high activity, light green indicates low activity. The response can be almost non-existent until a certain threshold is reached, whereafter the dramatically shifts in behaviour and settles in a different attractor (PTHrP). Another option is a gradual shift from one attractor to another, with the attractor slowly but steadily morphing into a different one, with no actual transition taking place at any time (IGF-I). A combination of these two situations is also possible, where the trends can also oppose each other, resulting in an ambivalent effect of the growth factor on certain genes (Wnt, BMP).

Specifically, we perturbed the six growth factors in the chondrocyte network from the Sox9 stable attractor. The results of perturbations of 1%, 5% and 10% to 50% are summarised in Table 2. As the algorithm randomly chooses nodes to update, simulations starting from the fixed perturbed state will not necessarily end up in the same attractor. Therefore, the upper number in each cell indicates the fraction of simulations that will resettle in the Sox9 attractor. The lower number indicates what activity the Sox9 node has in the perturbed attractor, the ‘wild type’ value being 0,9. This value is relevant since it may have phenotypic repercussions (e.g. more matrix protein production). The results show that qualitatively different responses can be delineated. In the case of PTHrP, the activity of Sox9 is not modulated until at a certain threshold value, the perturbation pushes the network out of the Sox9 attractor. Contrasting this threshold behaviour, perturbations in IGF cause a more gradual decrease (but still quite drastic in the 20 to 30% range) in Sox9 activity until it is quenched entirely. Here, the activity of Sox9 is modified without instantaneously pushing the network out of the Sox9 attractor. Wnt shows a combination of these two reactions, where the activity of Sox9 decreases gradually at first, until a switch to a different attractor ensues. For BMP a steady increase in Sox9 activity abruptly ends when the perturbation causes a transition to a different attractor, showing that the effect of a lower dose can be radically different from that of a higher one. Note that often a threshold (as high as 30% for IGF) must be passed before significant changes can be seen in the Sox9 activity, as only for Wnt and BMP the 1% perturbation entails a minor Sox9 activity change. Conceptually, this is shown in Fig 5. The advantage of the use of normalized continuous values is hence that they broaden the model’s scope to include predictions of qualitative dosage response. It should be noted that these qualitative responses are identical to the ones seen in the unmodified chondrocyte network, adhering to the rules presented above (see S2 Table).

A second major difference is the introduction of priority classes, in this case limited to two, allowing for a more straightforward breakdown of their contribution to model dynamics. To indicate their significance, we will investigate their influence on the canalisation of the Th network. For the Th network without priority classes, which boils down to using random asynchronous updating, the distribution of the Th1 vs the Th2 attractor is around 57% versus 43%. To assess the impact of the priority classes, we assigned a priority class to each interaction. The assigned priority class for each reaction is found in S3 Table. For the Th network with priority, the distribution is about 67% versus 33%, showing a relatively small impact on the canalisation analysis. Note that splitting into slow and fast control can entail a change in the control function, since it is effectively split into a slow and a fast part that are multiplied. As a control, we simulated the network with the altered control functions but without the separation into slow and fast parts (shown as result without priority in Table 3), effectively resorting to general asynchronous updating. This indeed showed that the alteration of the control function did not or only slightly affect the distribution (~0,3% difference).

**Table 3:** Results of the canalisation analysis for the Th network.

Attractor	Th1	Th2
**No priority (original)**	55,6% ± 0,5%	44,4% ± 0,5%
**No priority**	57,1% ± 1,0%	42,9% ± 1,0%
**Priority**	66,8% ± 0,9%	33,2% ± 0,9%
**GATA3 inhibition fast**	77,1% ± 0,8%	22,9% ± 0,8%
**T-bet inhibition fast**	60,2% ± 1,1%	39,8% ± 1,1%
**Both fast**	74,9% ± 0,1%	25,1% ± 0,1%

‘No priority (original)’ contains the results for the network where the control function is not adapted and reactions are not divided into priority classes. In ‘No priority’ this control function is adapted to be identical to the one used with priority classes included. All simulations were done in triplicate to supply a standard deviation.

Since the mutual repression of T-bet and GATA3 is a hypothesis [40], exact molecular mechanisms are unknown. In the above we have assumed the repression to be slow. Table 3 summarizes what the results would be if the repressions would be changed to fast, individually or both at the same time. The uncertainty on the priority class of the inhibitions results in a difference in the canalisation of the Th1 attractor from about 60% to 77%, a considerable difference that can determine which of the attractors is dominant. This shows that the introduction of priority classes can have a substantial impact.

The observed change is solely quantitative. For a model that is qualitative in nature, the obtained difference is perhaps not worth the added computational effort. That cannot be stated, however, when the change is qualitative. Though priority changes do not alter the attractors themselves, they can adversely affect its reachability. We illustrate this with a fictive example in the Th network, where we made the interactions that make up the GATA3 positive feedback (IL-4, IL-4R, STAT6 and GATA3, this includes negative feedback so that control functions are unchanged) fast, and all others interactions in the network slow. Thus these nodes are exclusively controlled by a fast variable, whereas all other nodes are determined by a slow variable. This shifts the equilibrium in favour of GATA3 to about 65%, but more importantly, 35% of random states end up in the Th0 state, while the Th1 state becomes unreachable.

The introduction of priority classes and normalized values in combination with additive logic hence has significant advantages in the above examples. However, these advantages have to be weighed against increased model complexity. Moreover, the advantages are not always present, as in some cases the extra features offer no, or relatively little, benefit over more basic models. For example the introduction of normalized values in the Th network does not change the effect of growth factors at all, mainly since dominant inhibitions prevent them from having an effect on the attractor. Hence in that case, a perturbation analysis in the Boolean framework will not differ qualitatively from one using normalized values. The same holds true for the influence of priority classes in the chondrocyte network. To illustrate this, we investigated the effect of a change in priority class for all nodes exclusively controlled by one priority class. The results can be found in S4 Table. None of these changes impacted the distribution by more than 10%, and the majority had a negligible effect.

## Discussion

This study highlights that extra features added to the Boolean framework give rise to a broader scope of model behaviour. Firstly, the introduction of continuous normalized values shows more diverse qualitative responses to growth factor perturbation. Secondly, the introduction of priority classes can affect the reachability of attractors. However, the extended scope must be balanced with limited availability of quantitative information, as models may keep to behaviour that the Boolean framework can capture as well.

As stated in the methods section, we employ an adapted form of general asynchronous updating, where each step an eligible node is selected to be updated randomly until a stable state is reached, rather than a fixed or synchronous updating scheme [56,57]. Since different updating mechanisms can lead to different behaviours and given a lack of information on reaction rates, we attempt to determine the properties of an ensemble of putative network dynamics. In an ensemble approach simulations of possible future states are generated with the aim of generating a good sample of the possible behaviour of a dynamical system [58,59]. Thus each simulation with specific updating order is one realization of the ensemble of networks with all possible reaction rates. The underlying idea is to discern what the possible range of behaviours is, rather than studying one specific realization of the dynamics. By doing a multitude of simulations we can hence get an estimate of the mainstay behaviour in the ensemble, and evaluate what range of behaviours are displayed under the set of possible discrete update sequences. While it would be theoretically possible to generate solutions for all possible update strategies, this approach is computationally intractable for more elaborate networks without the use of network reduction techniques [60].

Limited time information is added to the framework by dividing interactions into priority classes based on the expected rate of the underlying biological phenomenon. This division of the updating scheme in two priority classes equates to a quasi-steady state approach as we implicitly assume the fast variables to be in equilibrium as the slow variables evolve. The updating of nodes within the same priority class is random. Hence the time resolution of the model is improved by poising a balance between deterministic updating that requires more detailed time information and general asynchronous updating that requires none [11]. An important advantage in the introduction of priority classes is that they improve the biological plausibility in the ensemble of networks generated by the general asynchronous updating method, since it avoids the indiscriminate enumeration of all possible sequences of node updates, which includes many incompatible or unrealistic pathways [36]. Through the use of priority classes, we limit their spurious contribution to the ensemble’s behaviour. Moreover, this modification results in an augmentation of the model’s biological realism by clearly delineating interactions that change mRNA values and interactions influencing the concentration of the active protein. As a consequence a separate value for mRNA and protein is obtained, allowing the network to be more readily compared to results of common biological measurement techniques that rely on RNA (RNA-seq, microarrays, …).

The overall effect of the introduction of priority classes, as shown by the analysis of the chondrocyte network, could potentially be small in comparison to uncertainties that are incorporated by the weights, implicitly or explicitly (i.e. not all models have weights spelled out, but they are always there) and even more so by uncertainties on the type of the interactions [61] and the global topology of the network [62]. Nonetheless, the outliers in the results and the Th network do show that adding priority classes can have a large impact on the overall result, indicating the danger in ignoring ‘kinetic’ information.

The underlying assumption of the division of the model’s reactions into two priority classes is an ideal separation of the time scales on which these interactions take place. That is, the fast variables should reach equilibrium in a time frame where the slow variables only change marginally. This boils down to a quasi-steady state approach were we assume the fast reactions are always in equilibrium as the slow reactions evolve [63]. How well these assumptions are met in reality depends on the number of fast events that have to occur before (quasi-)equilibrium is reached and whether the fast processes are indeed (at least) a magnitude of order faster. A difficulty is thus found in the construction of the network, when processes are divided in the fast or slow category. Some processes are easy to categorize: phosphorylation events, lasting milliseconds [64], belong to the fast category while degradation of mRNA or proteins takes place on a scale of hours and hence belongs to the slow category [65]. Other processes, such as internalisation of growth factor-receptor complexes or nuclear shuttling of certain transcription factors do not fall squarely into either category. We have assigned all interactions that encompass transcriptional events or degradation processes to the slow category and all interactions that pertain to signalling events (including nuclear shuttling) to the fast one. This difficulty can be alleviated by including more categories, as was done in the apoptosis model of Schlatter et al [17].

The control functions employed in the framework are additive. Additive functions have shown to be capable of adequately simulating biological systems [66]. The use of additive functions assumes linear behaviour on the molecular scale, while it is often argued that many biological signalling systems behave in a nonlinear fashion. Of course, in larger networks additive control functions can still readily lead to emergent switch-like nonlinear behaviour, as a consequence of saturation in combination with topology and feedback loops. Using linear functions we can ensure that any nonlinear behaviour is a property of the system of interactions, rather than introduced through nonlinearities in the mathematics that simulate them [44]. At the same time, additive behaviour is also commonplace in biology and is often observed in, for example, the cellular response to growth factor stimulation [67]. A caveat is that additive responses on the population level may correspond to bimodal responses on the cell level [68]. Furthermore, the additive functions used here are applied in empirical methods such as network component analysis as well, further demonstrating their biological plausibility [69,70].

Fig 5 shows how the use of continuous variables allows examination of a dosage effect. Notably, in the chondrocyte network the effect of BMP signalling on the Sox9 phenotype is dosage-dependent. A low BMP signal is capable of increasing chondrocyte stability by increasing the activity of Sox9. Nonetheless, a stronger and protracted signal induces a phenotypic switch towards hypertrophy (shown as grey attractor in Fig 5). Indeed, BMP activity is more beneficial to the Runx2 related gene network than it is to the Sox9 transcriptional program. However, a low BMP signal is not strong enough to lift the inhibitions that Sox9 and related factors impose on the Runx2 differentiation program, and cannot disturb the positive feedback that stabilizes the Sox9 state. Specifically, the effects of BMP through its canonical pathway (and ERK) are initially being blocked by the activity of genes associated with Sox9 while other effects through p38 and Msx2 can increase the activity of Sox9. Note that a similar process occurs in the growth plate, where BMP activity progressively increases until hypertrophy [71]. Additionally, these findings agree with other reports in literature that endogenous BMP is required to maintain the cartilage phenotype and reinforces expression of the primary components of cartilage, collagen II and aggrecan [72]. On the other hand, the activation of Smad 1-5-8, the canonical BMP signalling pathway has been shown to result in hypertrophy [73]. A detailed study in the growth plate shows that low levels of BMP in the resting zone may help stave off differentiation, while intermediate levels in the proliferating zone would help the cells proliferate. Even farther down, high BMP signalling may contribute to hypertrophic differentiation [74].

From a methodological perspective, our use of additive control functions prevents having to make choices between implementing OR and AND gates to combine multiple signals, which are mostly arbitrary in the absence of information. An alternative to this approach could be to investigate an ensemble of networks with possible control functions, by for example varying the strength of the interactions, using different combinations of logical operators or probabilistic control functions [15]. However, the high number of signals to be combined in large networks leads to a combinatory explosion that renders this strategy computationally intractable if one wants to sample a significant fraction of the ensemble.

In the control functions the strength of interactions was assumed equal for all actors, in the absence of additional information. Sometimes information on, for example, the steady state is available allowing these to be tuned somewhat to reflect the data. A change in the importance of the interactions can skew the distribution of stable states in the canalisation analysis, though qualitative results are expected to be similar. For example, increasing the weight of any nodes’ outgoing edges’ impact will result in more influence of changes in that node’s activity on the stable state distribution and will also affect the ‘wild type’ canalization of stable states. Of course, the influence of any factor can be increased or decreased up to the point that its expression becomes dominant or inconsequential. In the former case this amounts to changing network topology by removing all other incoming links from nodes on which that particular node impacts, whereas the latter changes network topology by removing all of the node’s outgoing connections. Therefore it is recommended that any conclusions drawn from this generic framework should be subjected to a sensitivity analysis, for example by varying model assumptions.

There are several limitations to the additive approach presented here. The activities of complexes are represented by the product of the activities of its constituents, comparable to mass action kinetics (the activity of the complex is proportional to the number of collisions between its constituents). This representation has a few limitations, the first being that normalisation of the variables entails the inherent assumption that the concentrations of both constituents are the same order of magnitude. Secondly, it assumes one molecule of each constituent is present in the complex. Finally, the constituents don’t lose activity due to being holed up in the complex (no mass balance). This is only valid when a relatively low percentage of the constituents is in complex. Other formalisms, such as petri nets or mass action kinetics, may be better suited to model complexes [63].

Another caveat is that the examples discussed do not involve limit cycles. As such, the supplied implementation files (S2 File) do not detect limit cycles. Cycles may be detected numerically by comparing the updated state with a number of previous iterations. The introduction of priority classes presents an extra difficulty in this detection, since a limit cycle may also be present in the fast variables. Conceptually, this can be overcome by averaging over this ‘fast’ cycle before updating a new slow variable.

It is interesting to note that the framework presented here has qualitatively different attractors for the Th network from the ones obtained in Mendoza et al. [31]. This is primarily the consequence of the absence of ‘downward saturation’. The self-activation of T-bet can never lower the value of T-bet in our framework, whereas this occurs for low values in Mendoza’s framework. The shape of the activation function hence determines the stability (or rather, reachability) of this attractor. Generally, low values will, in the absence of other reactions, be saturated towards zero in the framework of Mendoza et al., ensuring the reachability of the trivial (Th0) state. In the absence of data on the exact kinetics of this self-activation no method deserves preference. Regardless, for low concentrations the continuum approach applied in both methods will tend to break down [44]. This discrepancy illustrates that the choice of framework represents an important assumption, and cautions against using a singular (generic or otherwise) framework to draw conclusions.

Compared to existing methods, our approach specifically applies additive logic and is tailored to information typically found in biological literature. As such, the presented framework is particularly suited for exploratory modelling of differentiation networks. Typically, these networks deal with growth factors that through a variety of signalling events influence a set of transcription factors that are needed to sustain a certain phenotype. In our experience, the type of interactions this involves (signalling events on the one hand and transcription on the other) lend themselves to division into two distinct priority classes. Moreover, the framework is of qualitative nature and geared towards systems where data is sparse. In the absence of combinatorial data, we employ an additive approach to deal with the multitude of pathways that converge on critical growth factors as a first step in exploring the system’s behaviour. The division of nodes into a fast and slow variable also helps in limiting the number of interactions that have to be combined in a single variable. The use of additive logic and normalisation of the variables can organically reveal dosage effects.

Somewhat paradoxically, our understanding of intracellular networks has progressed to a point that we can no longer comprehend them without resorting to mathematical models. Indeed, the fact that human intuition does not take dynamics into account, while these exploratory models do, is precisely their main contribution [16,75,76]. Many times a plausible topology is not easily unified with plausible dynamics, a fact quickly and precariously overlooked without explicit modelling. With that in mind, the challenge of simulating the dynamics is greater still than inferring topology, a far from trivial feat in itself [77]. The combinatorial explosion, already present in the multitude of candidate topologies, is further compounded by each topology having numerous different ways to play out dynamically. Nonetheless, even without fully resolving the myriad of possibilities, glancing on the dynamics can be instrumental in making a selection in topologies based on dynamic plausibility (or in practice, plausibility in a subset of dynamics).

Uncertainties on the priority classes, parameters and often also the network structure itself make predictions on the dynamic properties, e.g. canalisation, highly challenging, even if only qualitative. In these exploratory models, a key aspect to match the dynamics of the biological system is the topology [62]. Indeed, though some networks are remarkably stable to changes in interactions (such as the budding yeast cell cycle network [78]), an alteration of topology often entails different dynamics. Consequently, by adding or removing interactions, the dynamics of candidate networks can be investigated and optimized depending on the application [21,26,27,79]. Being exploratory models, it is clear that model parameters are often determined heuristically, accentuating that any conclusions derived from particular values are suspect. Consequently, only conclusions valid for a wider range of realistic values should be retained. This framework, incorporating increased and available time and semi-quantitative resolution, can help in substantiating the litmus test of dynamics to gene networks, firstly by excluding unlikely dynamics from the ensemble and secondly by refining falsifiable predictions on qualitative behaviour.

## Supporting Information

S1 Appendix Full list of equations for the chondrocyte network. (PDF)

S1 Fig Histogram of the first 5000 values (other than zero) of T-bet obtained by repeated random sampling. No values lower than 0,03 are detected. There is a clear trend for lower values to be less likely.
(PDF)

S1 File Chondrocyte model in SBML format. To capture the structure of the model presented here, each node is split into a slow and fast variable. Fast variables are identified by _PTM suffix. Slow variables have a _protein suffix. The final activity has no suffix. Fast reactions are identified by their reaction id. Inhibitions are identified by a negative sign in the kinetic law.
(XML)

S2 File MATLAB implementation files. AttractorThnetwork.m performs a random initialisation Monte Carlo analysis on the Th network without priority classes. AttractorThnetworkpriority.m does include priority. Attractorchondrocyte.m network performs a random initialisation Monte Carlo analysis on the chondrocyte network. Dosageeffect.m performs a perturbation analysis on the chondrocyte network.
(ZIP)

S1 Table The stable states of chondrocyte network. This table shows the attractors of the chondrocyte network. The three attractors are dubbed ‘None’, ‘Sox9’ and ‘Runx2’ representing the attractors where neither Sox9 or Runx2 is active, Sox9 is active and Runx2 is active, respectively. The first column gives the activity of the node. This activity is composed of the slow variable (second column), which gives the influence of the slow processes leading to protein formation, and the fast variable (third column), giving the influence of post translation modifications (PTMs).
(PDF)

S2 Table Results of perturbations for unmodified network. As can be seen in this table the results for Wnt, FGF, IGF and PTHrP are qualitatively the same. A difference arises in the qualitative response for BMP and Ihh due to the saturation of Sox9 activity at 1. However, it can be seen that the underlying unsaturated control function does show a similar dynamic for Sox9 activity.
(PDF)

S3 Table Allocation of the interactions to the 2 priority classes, i.e. fast or slow. Fast interactions consist of post translation modifications, receptor binding, and other interactions that take place in this time scale. Slow interactions include transcription, translation and degradation.
(PDF)

S4 Table The effect of a change in priority class for the chondrocyte network. S → F means the priority class was changed from fast to slow and vice versa. The third column gives the associated change in size of the Runx2 attractor basin.
(PDF)
