# Supplementary material for: A Semiquantitative Framework for Gene Regulatory Networks: Increasing the Time and Quantitative Resolution of Boolean Networks
Source: PLoS One. 2015 Jun 11;10(6):e0130033. doi: 10.1371/journal.pone.0130033 (PMC4489432; doi:10.1371/journal.pone.0130033)
Supplement: S1 Appendix — (PDF) [file pone.0130033.s001.pdf]

Full list of equations for the chondrocyte network.

For clarity's sake, each node is given a number, shown in the table below.

Node	Number
Wnt	1
Dsh	2
IGF-I	3
R-smad	4
Ihh	5
Gli2	6
β-catenin	7
Lef/Tcf	8
Runx2	9
Sox9	10
PTHrP	11
PPR	12
Col-X	13
PKA	14
MEF2C	15
FGF	16
FGFR3	17
STAT1	18
Smad complex	19
Col-II	20
Nkx3.2	21
ERK1/2	22
TGFβ	23
MMP13	24
Smad7	25
Smad3	26
FGFR1	27
ATF2	28
NFκβ	29
HDAC4	30
CCND1	31
Dlx5	32
BMP	33
p38	34
GSK3β	35
DC	36
PP2A	37
Akt	38

PI3K	39
Ets1	40
Ras	41
IGF-IR	42
Msx2	43
δ-EF1	44
ATF4	45
HIF-2α	46

Each node's activity z is determined by the product of its fast and slow variable ($z = z^f \times z^s$). The saturation factor is set at 2/3. Therefore $s_i = 2 * \frac{saturation\,factor}{i}$ where i is the number of excess positive interactions.

$$z_1^f(t+1) = 1$$

$$z_1^s(t+1) = z_6(t) + z_7(t)z_8(t) - z_{43}(t)$$

$$z_2^f(t+1) = z_1(t) - 0.5z_4(t)$$

$$z_2^s(t+1) = 1$$

$$z_3^f(t+1) = 1$$

$$z_3^s(t+1) = z_{14}(t)$$

$$z_4^f(t+1) = z_{33}(t) - z_{25}(t)$$

$$z_4^s(t+1) = 1$$

$$z_5^f(t+1) = 1$$

$$z_5^s(t+1) = \left(z_9(t) + z_{19}(t) + z_{29}(t) + z_{45}(t) - z_{44}(t)\right)s_2$$

$$z_6^f(t+1) = \frac{2}{3}z_5(t)$$

$$z_6^s(t+1) = 1$$

$$z_7^f(t+1) = 1 - z_{36}(t)$$

$$z_7^s(t+1) = 1$$

$$z_8^f(t+1) = z_7(t) - z_{10}(t)$$

$$z_8^s(t+1) = \left(1 + z_7(t) + z_9(t)\right)s_3$$

$$z_9^f(t+1) = z_{38}(t) + z_{22}(t) + z_{32}(t) + z_{14}(t) - z_{10}(t) - z_{26}(t)z_{30}(t) - z_{31}(t) - z_{43}(t)$$

$$z_9^s(t+1) = \left(z_{46}(t) + z_8(t) + z_9(t) + z_{15}(t) + z_{32}(t) - z_{21}(t)z_{19}(t) - z_{43}(t)\right)s_3$$

$$z_{10}^{f}(t+1) = z_{14}(t) + z_{26}(t) - z_{7}(t) - z_{9}(t)$$

$$z_{10}^{s}(t+1) = \big(z_{14}(t) + z_{34}(t) + z_{29}(t) + z_{10}(t) + z_{21}(t) - z_{29}(t)\big)s_4$$

$$z_{11}^{f}(t+1) = 1$$

$$z_{11}^{s}(t+1) = \big(z_{6}(t)z_{10}(t) + z_{10}(t) + z_{14}(t) + z_{26}(t)\big)s_4$$

$$z_{12}^{f}(t+1) = z_{11}(t)$$

$$z_{12}^{s}(t+1) = \big(z_{6}(t) + z_{10}(t) + z_{19}(t) - z_{18}(t)\big)s_2$$

$$z_{13}^{f}(t+1) = 1$$

$$z_{13}^{s}(t+1) = \big(z_{9}(t) + z_{4}(t) + z_{15}(t) - z_{14}(t)\big)s_2$$

$$z_{14}^{f}(t+1) = \big(z_{3}(t) + z_{12}(t)\big)s_2$$

$$z_{14}^{s}(t+1) = 1$$

$$z_{15}^{f}(t+1) = z_{19}(t) + z_{34}(t) - z_{30}(t) - z_{26}(t)$$

$$z_{15}^{s}(t+1) = \big(z_{9}(t) + z_{19}(t)\big)s_2$$

$$z_{16}^{f}(t+1) = 1$$

$$z_{16}^{s}(t+1) = \big(z_{7}(t) + z_{9}(t)\big)s_2$$

$$z_{17}^{f}(t+1) = z_{16}(t)$$

$$z_{17}^{s}(t+1) = z_{10}(t)$$

$$z_{18}^{f}(t+1) = \frac{2}{3}\left(\frac{2}{3}z_{17}(t) + \frac{2}{3}z_{27}(t)\right) + \frac{1}{3}z_{42}(t) - z_{19}(t)$$

$$z_{18}^{s}(t+1) = 1$$

$$z_{19}^{f}(t+1) = z_{4}(t) - z_{22}(t)$$

$$z_{19}^{s}(t+1) = 1$$

$$z_{20}^{f}(t+1) = 1$$

$$z_{20}^{s}(t+1) = \big(z_{10}(t) + z_{27}(t) + z_{21}(t)\big)s_3$$

$$z_{21}^{f}(t+1) = 1$$

$$z_{21}^{s}(t+1) = \big(z_{10}(t) + z_{12}(t)\big)s_2$$

$$z_{22}^{f}(t+1) = z_{41}(t) - z_{37}(t)$$

$$z_{22}^{s}(t+1) = 1$$

$$z_{23}^{f}(t+1) = 1$$

$$z_{23}^{s}(t+1) = z_6(t)$$

$$z_{24}^{f}(t+1) = 1$$

$$z_{24}^{s}(t+1) = \big(z_9(t) + z_{29}(t) + z_{46}(t) + z_9(t)z_{34}(t)z_{46}(t) - z_6(t)\big)\frac{1}{4}$$

$$z_{25}^{f}(t+1) = 1$$

$$z_{25}^{s}(t+1) = \big(z_{18}(t) + z_{19}(t) + z_{26}(t) + 2z_{29}(t)\big)s_5$$

$$z_{26}^{f}(t+1) = z_{23}(t) - 0.5z_{25}(t) - 0.5s_2\big(z_{22}(t) + z_{31}(t)\big)$$

$$z_{26}^{s}(t+1) = 1$$

$$z_{27}^{f}(t+1) = z_{16}(t) - 0.5z_{19}(t)$$

$$z_{27}^{s}(t+1) = z_9(t)$$

$$z_{28}^{f}(t+1) = z_{26}(t)z_{34}(t)$$

$$z_{28}^{s}(t+1) = z_{26}(t)$$

$$z_{29}^{f}(t+1) = \big(z_{27}(t) + z_{38}(t)\big)s_2$$

$$z_{29}^{s}(t+1) = 1$$

$$z_{30}^{f}(t+1) = z_{37}(t) - z_{35}(t)$$

$$z_{30}^{s}(t+1) = 1$$

$$z_{31}^{f}(t+1) = 1 - 0.5\big(0.5z_{35}(t) + z_{18}(t)\big)$$

$$z_{31}^{s}(t+1) = \big(z_{29}(t) + z_6(t) + z_{14}(t)\big)s_3$$

$$z_{32}^{f}(t+1) = z_{34}(t) + z_{19}(t) - z_{43}(t)$$

$$z_{32}^{s}(t+1) = z_{15}(t) + z_{19}(t) - z_{26}(t) - z_{43}(t)$$

$$z_{33}^{f}(t+1) = 1$$

$$z_{33}^{s}(t+1) = \big(z_6(t) + z_{29}(t)\big)s_2$$

$$z_{34}^{f}(t+1) = \big(z_{23}(t) + z_{33}(t)\big)s_2$$

$$z_{34}^{s}(t+1) = 1$$

$$z_{35}^{f}(t+1) = 1 - \big(z_{14}(t) - z_{37}(t)\big)z_{22}(t)$$

$$z_{35}^{s}(t+1) = 1$$

$$z_{36}^{f}(t+1) = \big(1 - z_2(t)\big)min(1.5 - z_{22}(t), 1)$$

$$z_{36}^{s}(t+1) = 1$$

$$z_{37}^{f}(t+1) = z_{14}(t)$$

$$z_{37}^{s}(t+1) = 1$$

$$z_{38}^{f}(t+1) = z_{39}(t) - 0.5z_{37}(t)$$

$$z_{38}^{s}(t+1) = z_9(t)$$

$$z_{39}^{f}(t+1) = \big(z_{41}(t) + z_{42}(t)\big)s_2$$

$$z_{39}^{s}(t+1) = z_9(t)$$

$$z_{40}^{f}(t+1) = \big(z_{22}(t) + z_{35}(t)\big)s_2$$

$$z_{40}^{s}(t+1) = z_{26}(t)$$

$$z_{41}^{f}(t+1) = \big(z_1(t) + z_{33}(t) + z_{17}(t) + z_{27}(t)\big)s_3$$

$$z_{41}^{s}(t+1) = 1$$

$$z_{42}^{f}(t+1) = z_3(t)$$

$$z_{42}^{s}(t+1) = z_{23}(t) + z_{29}(t) - z_{18}(t)$$

$$z_{43}^{f}(t+1) = 1 - z_{32}(t)$$

$$z_{43}^{s}(t+1) = z_{19}(t) + z_{26}(t) - z_{32}(t)$$

$$z_{44}^{f}(t+1) = 1$$

$$z_{44}^{s}(t+1) = \big(z_{29}(t) + z_{40}(t)\big)s_2$$

$$z_{45}^{f}(t+1) = \big(z_{14}(t) + z_{22}(t)\big)s_2$$

$$z_{45}^{s}(t+1) = 1$$

$$z_{46}^{f}(t+1) = 1$$

$$z_{46}^{s}(t+1) = z_{29}(t)$$

Full list of equations for the T helper cell network.

For clarity's sake, each node is given a number, shown in the table below.

Node	Number
TCR	1
NFAT	2
IFN-β	3
IFN-βR	4
IL-18	5
IL-18R	6
IRAK	7
SOCS1	8
IL-12	9
IL-12R	10
STAT4	11
T-bet	12
IFN-γ	13
IFN-γR	14
JAK1	15
STAT1	16
IL-4	17
IL-4R	18
STAT6	19
GATA3	20
IL-10	21
IL-10R	22
STAT3	23

Each node's activity z is determined by the product of its fast and slow variable ($z = z^f \times z^s$). The saturation factor is set at 2/3. Therefore $s_i = 2 * \frac{saturation\ factor}{i}$ where i is the number of excess positive interactions.

$$z_1^f(t+1) = 1$$

$$z_1^s(t+1) = 0/z_1(t)$$

$$z_2^f(t+1) = z_1(t)$$

$$z_2^s(t+1) = 1$$

$$z_3^f(t+1) = 1$$

$$z_3^s(t+1) = 0/z_3(t)$$

$$z_4^f(t+1) = z_3(t)$$

$$z_4^s(t+1) = 1$$

$$z_5^f(t+1) = 1$$

$$z_5^s(t+1) = 0/z_5(t)$$

$$z_6^f(t+1) = z_5(t) - z_{19}(t)$$

$$z_6^s(t+1) = 1$$

$$z_7^f(t+1) = z_6(t)$$

$$z_7^s(t+1) = 1$$

$$z_8^f(t+1) = \big(z_{12}(t) + z_{16}(t)\big)s_2$$

$$z_8^s(t+1) = 1$$

$$z_9^f(t+1) = 1$$

$$z_9^s(t+1) = 0/z_9(t)$$

$$z_{10}^f(t+1) = z_9(t) - z_{19}(t)$$

$$z_{10}^s(t+1) = 1$$

$$z_{11}^f(t+1) = z_{10}(t) - z_{20}(t)$$

$$z_{11}^s(t+1) = 1$$

$$z_{12}^f(t+1) = 1$$

$$z_{12}^s(t+1) = \big(z_{12}(t) + z_{16}(t)\big)\big(1 - z_{20}(t)\big)$$

$$z_{13}^f(t+1) = 1$$

$$z_{13}^s(t+1) = \big(z_1(t) + z_7(t) + z_{11}(t) + z_{12}(t) - z_{23}(t)\big)s_3$$

$$z_{14}^f(t+1) = z_{13}(t)$$

$$z_{14}^s(t+1) = 1$$

$$z_{15}^f(t+1) = 1$$

$$z_{15}^s(t+1) = z_{14}(t) - z_8(t)$$

$$z_{16}^f(t+1) = 1$$

$$z_{16}^{s}(t+1) = \left(z_{15}(t) + z_4(t)\right)s_2$$

$$z_{17}^{f}(t+1) = 1$$

$$z_{17}^{s}(t+1) = z_{20}(t) - z_{16}(t)$$

$$z_{18}^{f}(t+1) = z_{17}(t) - z_8(t)$$

$$z_{18}^{s}(t+1) = 1$$

$$z_{19}^{f}(t+1) = z_{18}(t)$$

$$z_{19}^{s}(t+1) = 1$$

$$z_{20}^{f}(t+1) = 1$$

$$z_{20}^{s}(t+1) = \left(z_{19}(t) + z_{20}(t)\right)\left(1 - z_{12}(t)\right)$$

$$z_{21}^{f}(t+1) = 1$$

$$z_{21}^{s}(t+1) = z_{20}(t)$$

$$z_{22}^{f}(t+1) = z_{21}(t)$$

$$z_{22}^{s}(t+1) = 1$$

$$z_{23}^{f}(t+1) = z_{22}(t)$$

$$z_{23}^{s}(t+1) = 1$$

Here the nodes TCR, IFN-β and IL-18 can be either zero or stay at their initial value, depending on whether they are regarded as input or not. In the simulations included in the paper, these nodes are set at zero. For the Monte Carlo analysis with random initialisation, this amounts to have an initial activity that is removed after updating, simulating degradation of the growth factor.

Comparison of stable states

We compare the stable states of the Th network detected by the random initialisation Monte Carlo analysis to those identified in Mendoza et al and Mendoza et al. The stable states are given in the following table:

**Node**	**TCR**	**NFAT**	**IFN-β**	**IFN-βR**	**IL-18**	**IL-18R**	**IRAK**	**SOCS1**
	**Th1 (T-bet)**							
**Additive**	0	0	0	0	0	0	0	x/2
**Mendoza Boolean**	0	0	0	0	0	0	0	1
**Mendoza ODE**	0	0	0	0	0	0	0	0,89479
	**Th2 (GATA3)**							
**Additive**	0	0	0	0	0	0	0	0
**Mendoza Boolean**	0	0	0	0	0	0	0	0
**Mendoza ODE**	0	0	0	0	0	0	0	0
**Node**	**IL-12**	**IL-12R**	**STAT4**	**T-bet**	**IFN-γ**	**IFN-γR**	**JAK1**	**STAT1**
	**Th1 (T-bet)**							
**Additive**	0	0	0	x	x/3	x/3	0	0
**Mendoza Boolean**	0	0	0	1	1	1	0	0
**Mendoza ODE**	0	0	0	0,89479	0,71443	0,9719	0,00489	0,00051
	**Th2 (GATA3)**							
**Additive**	0	0	0	0	0	0	0	0
**Mendoza Boolean**	0	0	0	0	0	0	0	0
**Mendoza ODE**	0	0	0	0	0	0	0	0
**Node**	**IL-4**	**IL-4R**	**STAT6**	**GATA3**	**IL-10**	**IL-10R**	**STAT3**	
	**Th1 (T-bet)**							
**Additive**	0	0	0	0	0	0	0	
**Mendoza Boolean**	0	0	0	0	0	0	0	
**Mendoza ODE**	0	0	0	0	0	0	0	
	**Th2 (GATA3)**							
**Additive**	1	1	1	1	1	1	1	
**Mendoza Boolean**	1	1	1	1	1	1	1	
**Mendoza ODE**	1	1	1	1	1	1	1	

Comparison of stable states. x can in principle be any value in [0,1]. Mendoza Boolean refers to stable states for the Boolean version of the Th network in Mendoza and Xenarios [1]. Mendoza ODE refers to stable states of the network in the continuous framework of Mendoza and Xenarios [1]. Additive refers to the variant presented in this paper. The stable states for Th1 and Th2 are shown. In the 'None' state, all nodes are zero in all variants. The stable states are qualitatively similar in all three networks as all nodes are correspondingly active/ inactive for both Th1 and Th2 in all frameworks. JAK1 and STAT1 constitute a minor exception as they show some activity (0,5% and 0,005 % respectively) in the continuous framework of Mendoza and Xenarios [1], but none in the Boolean and additive versions.

Reference List

1. Mendoza L, Xenarios I (2006) A method for the generation of standardized qualitative dynamical systems of regulatory networks. Theoretical Biology and Medical Modelling 3: 13. 10.1186/1742-4682-3-13.
