# Supplementary material for: A Semiquantitative Framework for Gene Regulatory Networks: Increasing the Time and Quantitative Resolution of Boolean Networks
Source: PLoS One. 2015 Jun 11;10(6):e0130033. doi: 10.1371/journal.pone.0130033 (PMC4489432; doi:10.1371/journal.pone.0130033)

S1 Fig. Histogram of the first 5000 values (other than zero) of T-bet obtained by repeated random sampling. No values lower than 0,03 are detected. There is a clear trend for lower values to be less likely.

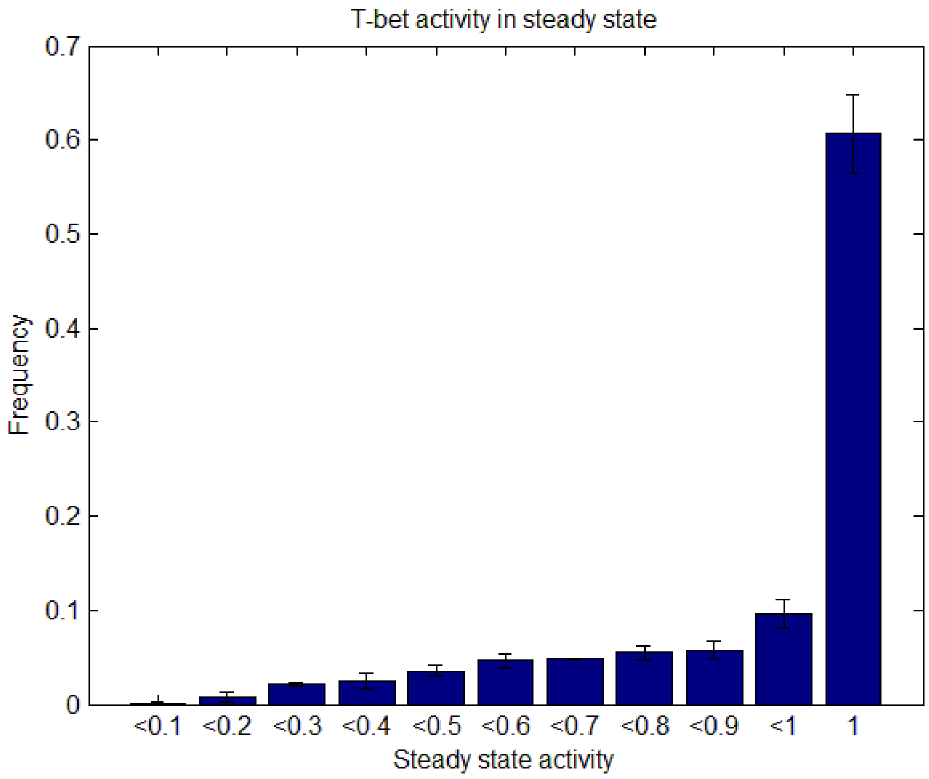

Supplement: S1 Fig — No values lower than 0,03 are detected. There is a clear trend for lower values to be less likely. (PDF) [file pone.0130033.s002.pdf]
