# Supplementary material for: A Semiquantitative Framework for Gene Regulatory Networks: Increasing the Time and Quantitative Resolution of Boolean Networks
Source: PLoS One. 2015 Jun 11;10(6):e0130033. doi: 10.1371/journal.pone.0130033 (PMC4489432; doi:10.1371/journal.pone.0130033)
Supplement: S1 Table — This table shows the attractors of the chondrocyte network. The three attractors are dubbed ‘None’, ‘Sox9’ and ‘Runx2’ representing the attractors where neither Sox9 or Runx2 is active, Sox9 is active and Runx2 is active, respectively. The first column gives the activity of the node. This activity is composed of the slow variable (second column), which gives the influence of the slow processes leading to protein formation, and the fast variable (third column), giving the influence of post translation modifications (PTMs). (PDF) [file pone.0130033.s005.pdf]

S1 Table. The stable states of chondrocyte network. This table shows the attractors of the chondrocyte network. The three attractors are dubbed 'None', 'Sox9' and 'Runx2' representing the attractors where neither Sox9 or Runx2 is active, Sox9 is active and Runx2 is active, respectively. The first column gives the activity of the node. This activity is composed of the slow variable (second column), which gives the influence of the slow processes leading to protein formation, and the fast variable (third column), giving the influence of post translation modifications (PTMs).

Node	Wnt	Dsh	IGF-I	R-smad	Ihh	Gli2	β-catenin	Lef/Tcf	Runx2	Sox9	PTHrP	PPR	Col-X	PKA	MEF2C	FGF
	None															
Activity	0,00	0,00	0,00	0,00	0,00	0,00	0,00	0,00	0,00	0,00	0,00	0,00	0,00	0,00	0,00	0,00
PTM	1,00	0,00	1,00	0,00	1,00	0,00	0,00	0,00	0,00	0,00	1,00	0,00	1,00	0,00	0,00	1,00
Protein	0,00	1,00	0,00	1,00	0,00	1,00	1,00	0,44	0,00	0,00	0,00	0,00	0,00	1,00	0,00	0,00
	Sox9															
Activity	0,29	0,21	1,00	0,16	0,52	0,35	0,21	0,00	0,00	0,90	0,77	0,72	0,00	1,00	0,05	0,14
PTM	1,00	0,21	1,00	0,16	1,00	0,35	0,21	0,00	0,00	0,90	1,00	0,77	1,00	1,00	0,44	1,00
Protein	0,29	1,00	1,00	1,00	0,52	1,00	1,00	0,54	0,00	1,00	0,77	0,93	0,00	1,00	0,11	0,14
	Runx2															
Activity	1,00	0,83	0,00	0,35	1,00	0,67	0,91	0,91	1,00	0,00	0,00	0,00	1,00	0,00	0,67	1,00
PTM	1,00	0,83	1,00	0,35	1,00	0,67	0,91	0,91	1,00	0,00	1,00	0,00	1,00	0,00	1,00	1,00
Protein	1,00	1,00	0,00	1,00	1,00	1,00	1,00	1,00	1,00	0,33	0,00	0,15	1,00	1,00	0,67	1,00

Node	FGFR3	STAT1	Smadcomplex	Col-II	Nkx3.2	ERK1/2	TGFβ	MMP13	Smad7	Smad3	FGFR1	ATF2	NFκβ	HDAC4	CCND1
	None														
Activity	0,00	0,00	0,00	0,00	0,00	0,00	0,00	0,00	0,00	0,00	0,00	0,00	0,00	0,00	0,00
PTM	0,00	0,00	0,00	1,00	1,00	0,00	1,00	1,00	1,00	0,00	0,00	0,00	0,00	0,00	0,75
Protein	0,00	1,00	1,00	0,00	0,00	1,00	0,00	0,00	0,00	1,00	0,00	0,00	1,00	1,00	0,00
	Sox9														
Activity	0,12	0,01	0,16	1,00	1,00	0,00	0,35	0,00	0,07	0,10	0,00	0,00	0,00	0,00	0,61
PTM	0,14	0,01	0,16	1,00	1,00	0,00	1,00	1,00	1,00	0,10	0,06	0,04	0,00	0,00	0,75
Protein	0,90	1,00	1,00	1,00	1,00	1,00	0,35	0,00	0,07	1,00	0,00	0,10	1,00	1,00	0,82
	Runx2														
Activity	0,00	0,44	0,00	1,00	0,00	1,00	0,67	0,83	0,65	0,00	1,00	0,00	1,00	0,00	0,16
PTM	1,00	0,44	0,00	1,00	1,00	1,00	1,00	1,00	1,00	0,00	1,00	0,00	1,00	0,00	0,53
Protein	0,00	1,00	1,00	1,00	0,00	1,00	0,67	0,83	0,65	1,00	1,00	0,00	1,00	1,00	0,30

Node	Dlx5	BMP	p38	GSK3β	DC	PP2A	Akt	PI3K	Ets1	Ras	IGF-1R	Msx2	δ-EF1	ATF4	HIF-2α
	None														
Activity	0,00	0,00	0,00	1,00	1,00	0,00	0,00	0,00	0,00	0,00	0,00	0,00	0,00	0,00	0,00
PTM	0,00	1,00	0,00	1,00	1,00	0,00	0,00	0,00	0,67	0,00	0,00	1,00	1,00	0,00	1,00
Protein	0,00	0,00	1,00	1,00	1,00	1,00	0,00	0,00	0,00	1,00	0,00	0,00	0,00	1,00	0,00
	Sox9														
Activity	0,00	0,23	0,38	1,00	0,79	1,00	0,00	0,00	0,07	0,28	0,34	0,26	0,05	0,67	0,00
PTM	0,28	1,00	0,38	1,00	0,79	1,00	0,00	0,41	0,67	0,28	1,00	1,00	1,00	0,67	1,00
Protein	0,00	0,23	1,00	1,00	1,00	1,00	0,00	0,00	0,10	1,00	0,34	0,26	0,05	1,00	0,00
	Runx2														
Activity	0,67	1,00	1,00	1,00	0,09	0,00	0,67	0,67	0,00	1,00	0,00	0,00	0,67	0,67	1,00
PTM	1,00	1,00	1,00	1,00	0,09	0,00	0,67	0,67	1,00	1,00	0,00	0,33	1,00	0,67	1,00
Protein	0,67	1,00	1,00	1,00	1,00	1,00	1,00	1,00	0,00	1,00	1,00	0,00	0,67	1,00	1,00
