# Supplementary material for: A Semiquantitative Framework for Gene Regulatory Networks: Increasing the Time and Quantitative Resolution of Boolean Networks
Source: PLoS One. 2015 Jun 11;10(6):e0130033. doi: 10.1371/journal.pone.0130033 (PMC4489432; doi:10.1371/journal.pone.0130033)
Supplement: S2 Table — As can be seen in this table the results for Wnt, FGF, IGF and PTHrP are qualitatively the same. A difference arises in the qualitative response for BMP and Ihh due to the saturation of Sox9 activity at 1. However, it can be seen that the underlying unsaturated control function does show a similar dynamic for Sox9 activity. (PDF) [file pone.0130033.s006.pdf]

S2 Table. Results of perturbations for unmodified network. As can be seen in this table the results for Wnt, FGF, IGF and PTHrP are qualitatively the same. A difference arises in the qualitative response for BMP and Ihh due to the saturation of Sox9 activity at 1. However, it can be seen that the underlying unsaturated control function does show a similar dynamic for Sox9 activity.

**Sign:**	**+**	**+**	**+**	**+**	**-**	**-**
**Factor:**	**Wnt**	**Ihh**	**FGF**	**BMP**	**IGF**	**PTHrP**
**1%**	1	1	1	1	1	1
	100%	100%	100%	100%	100%	100%
**5%**	1	1	1	1	1	1
	100%	100%	100%	100%	100%	100%
**10%**	1	1	1	1	1	1
	100%	100%	100%	100%	100%	100%
**20%**	1	1	1	1	1	1
	98%	100%	100%	100%	100%	100%
**30%**	0,95 ± 0,015	1	1	1	1	1
	88%	100%	100%	100%	100%	100%
**40%**	0	1	0,99 ± 0,01	1	1	0
	0	100%	100%	100%	14%	0%
**50%**	0	1	0,93 ± 0,025	1	1	0
	0	100%	100%	100%	9%	0%
**90%**	0	1	0	0,42 ± 0,06	0	0
	0%	100%	0%	100%	0,20%	0%
