# Supplementary material for: A Semiquantitative Framework for Gene Regulatory Networks: Increasing the Time and Quantitative Resolution of Boolean Networks
Source: PLoS One. 2015 Jun 11;10(6):e0130033. doi: 10.1371/journal.pone.0130033 (PMC4489432; doi:10.1371/journal.pone.0130033)
Supplement: S3 Table — Fast interactions consist of post translation modifications, receptor binding, and other interactions that take place in this time scale. Slow interactions include transcription, translation and degradation. (PDF) [file pone.0130033.s007.pdf]

S3 Table. Allocation of the interactions to the 2 priority classes, i.e. fast or slow. Fast interactions consist of post translation modifications, receptor binding, and other interactions that take place in this time scale. Slow interactions include transcription, translation and degradation.

Originating node	Receiving node	Priority
NFAT	TCR	fast
IFN-β	IFN-βR	fast
IL-18	IL-18R	fast
STAT6	IL-18R	fast
IL-18R	IRAK	fast
T-bet	SOCS1	fast
STAT1	SOCS1	fast
IL-12	IL-12R	fast
STAT6	IL-12R	fast
IL-12R	STAT4	fast
GATA3	STAT4	fast
IFN-γ	IFN-γR	fast
IL-4	IL-4R	fast
SOCS1	IL-4R	fast
IL-10	IL-10R	fast
IL-10R	STAT3	fast
T-bet	T-bet	slow
STAT1	T-bet	slow
GATA3	T-bet	slow
NFAT	IFN-γ	slow
IRAK	IFN-γ	slow
STAT4	IFN-γ	slow
T-bet	IFN-γ	slow
STAT3	IFN-γ	slow
IFN-γR	JAK1	slow
SOCS1	JAK1	slow
JAK1	STAT1	slow
IFN-βR	STAT1	slow
GATA3	IL-4	slow
STAT1	IL-4	slow
STAT6	GATA3	slow
GATA3	GATA3	slow
T-bet	GATA3	slow
GATA3	IL-10	slow
