# Supplementary material for: A Semiquantitative Framework for Gene Regulatory Networks: Increasing the Time and Quantitative Resolution of Boolean Networks
Source: PLoS One. 2015 Jun 11;10(6):e0130033. doi: 10.1371/journal.pone.0130033 (PMC4489432; doi:10.1371/journal.pone.0130033)
Supplement: S4 Table — S → F means the priority class was changed from fast to slow and vice versa. The third column gives the associated change in size of the Runx2 attractor basin. (PDF) [file pone.0130033.s008.pdf]

S4 Table. The effect of a change in priority class for the chondrocyte network. S → F means the priority class was changed from fast to slow, F → S indicates a change from slow to fast. The third column gives the associated change in the size of the Runx2 attractor basin.

Node name	Change	Runx2 attractor
**Wnt**	S → F	0,3%
**Dsh**	F → S	-1,1%
**IGF-I**	S → F	-0,8%
**R-smad**	F → S	7,7%
**Ihh**	S → F	-0,1%
**Gli2**	F → S	1,0%
**β-catenin**	F → S	-0,7%
**PKA**	F → S	1,0%
**Smadcomplex**	F → S	8,6%
**ERK1/2**	F → S	3,1%
**TGFβ**	S → F	-3,6%
**Smad7**	S → F	-0,8%
**Smad3**	F → S	-7,4%
**NFκβ**	F → S	3,3%
**HDAC4**	F → S	-3,2%
**BMP**	S → F	4,0%
**p38**	F → S	-8,8%
**GSK3β**	F → S	-2,8%
**DC**	F → S	-2,4%
**PP2A**	F → S	1,2%
**Ras**	F → S	-1,2%
**δEF-1**	S → F	-0,8%
**ATF4**	F → S	-1,2%
**HIF-2α**	S → F	-1,0%
